# Spontaneous Resolution of Follicular Lymphoma in an Elderly Caucasian Patient Undergoing Uninvolved Radiation Therapy for Recurrent Merkel Cell Carcinoma: A Case Report

**DOI:** 10.7759/cureus.88033

**Published:** 2025-07-15

**Authors:** Sina Foroutanjazi, Lacey McIntosh, Tasneem Ali, Dori Goldberg

**Affiliations:** 1 Dermatology, UMass Memorial Medical Center, Worcester, USA; 2 Radiology, UMass Memorial Medical Center, Worcester, USA; 3 Oncology, UMass Memorial Medical Center, Worcester, USA

**Keywords:** abscopal effect, follicular lymphoma, merkel cell cancer, radiation therapy, skin cancer recurrence

## Abstract

Merkel cell carcinoma (MCC) is a rare, aggressive cutaneous malignancy with an annual incidence rate of less than one case per 100,000 person-years in the United States, up to 59% of which have a reported risk of loco-regional and distant metastasis. While wide local excision with sentinel lymph node biopsy is the gold standard for the initial management of localized MCC, adjuvant radiation therapy is commonly utilized as a supplemental treatment modality for these patients as well. Here we present the case of a Caucasian woman in her 90s with recurrent MCC and concurrent grade 3A follicular lymphoma who received 6700 cGy of radiation therapy over two months for her recurrent MCC, which led to complete clearance of her uninvolved-site follicular lymphoma likely via an abscopal effect. While its exact mechanism needs to be further studied, the abscopal effect is thought to be a process in which radiation therapy of a malignant proliferation leads to destruction of the same malignancy in uninvolved sites. However, to our knowledge, the resolution of uninvolved-site follicular lymphoma in response to radiation therapy of a different malignancy such as MCC is not previously reported.

## Introduction

Merkel cell carcinoma (MCC) is a rare but highly aggressive form of cutaneous neuroendocrine cancer with an annual incidence of less than one case per 100,000 person-years and a median age of onset of 75 in the United States. MCC is strongly associated with infection with the Merkel cell polyomavirus, which is seen in up to 80% of the cases, and, less commonly, can arise secondary to the cumulative carcinogenic effect of chronic exposure to ultraviolet radiation [1,2]. MCC affects the head and neck sites in around 50% of the cases, followed by the extremities and the trunk in around 35% and 15% of the cases, respectively [3,4]. Immunohistochemical staining (IHC) for cytokeratin 20 (CK20) is highly sensitive for the diagnosis of MCC, with sensitivities reported to be 78%-91% in the current literature [5,6]. Negative thyroid transcription factor 1 (TTF-1) in the setting of positive CK20 is particularly sensitive in detecting metastatic MCC, although diagnostic challenges exist for rare cases of CK20-negative and TTF-1-positive MCC [6].

The aggressive nature of MCC is typically associated with a high rate of loco-regional or distant metastases, with previous reports showing between 19% and 59% risk of metastasis to the non-regional lymph nodes, skin, liver, bone, pancreas, lung, or brain [7,8]. Previous studies have shown sentinel lymph node (SLN) biopsy in patients with clinically node-negative localized MCC to be positive for malignancy in up to 38% of cases [5].

According to the most recent National Comprehensive Cancer Network and American Joint Committee on Cancer guidelines, the gold standard initial treatment for localized MCC is wide local excision (WLE) with SLN biopsy. While there are no clear guidelines regarding dosing and duration of radiation therapy (RT) for treatment of MCC, and, due to its low incidence, the effect of RT with respect to progress-free and disease-free survival in these patients is lacking, adjuvant RT is generally recommended for all stages of localized MCC [5]. Treatment modalities for the management of primary or metastatic MCC further include immune checkpoint inhibitors such as avelumab, a Food and Drug Administration (FDA)-approved programmed death-ligand inhibitor, pembrolizumab or nivolumab, which in the setting of MCC’s known immune susceptibility have become the treatments of choice in the management of advanced MCC. Other options such as cytotoxic chemotherapy are typically reserved for refractory cases [9-11].

Unlike MCC, which is an aggressive malignancy that tends to respond well to RT, a more indolent, similarly rare hematological malignancy affecting the elderly is follicular lymphoma, which shows a favorable response to this treatment modality as well. Follicular lymphoma is a non-Hodgkin's B-cell germinal center neoplasm commonly related to malignant transformation of precursor B-cells carrying the t(14;18) chromosomal translocation. It has an incidence rate of two to four per 100,000 person-years and a median age of 65 in the United States [12]. The treatment for follicular lymphoma is based on the tumor burden, tumor stage and characteristics, as well as prior response to treatments, and may include RT in limited or low-burden disease (stage I or II), or combination chemotherapy or targeted immunotherapy in a more advanced disease [13-15]. 

While destruction of a cancerous growth at the site of radiation is an expected outcome of RT, reports of resolution of distant lesions of the same malignancy in response to uninvolved site RT are available in the literature, which describe a process termed the abscopal effect. This is thought to occur in response to activation of the immune system upon exposure to the antigens that originated from the original cancerous lesion upon RT. Highly immunogenic cancers with a low immune evasion strategy are thought to be more likely to demonstrate this phenomenon compared to their more indolent, less immunogenic counterparts [13,15-18]. The resolution of a cancerous lesion in response to uninvolved site RT of a different cancer, however, is not previously reported.

Here we present the case of an elderly Caucasian patient with recurrent MCC and new-onset follicular lymphoma who underwent targeted RT for her MCC and was found to have complete metabolic and anatomic resolution of her follicular lymphoma.

## Case presentation

**Figure 1:**
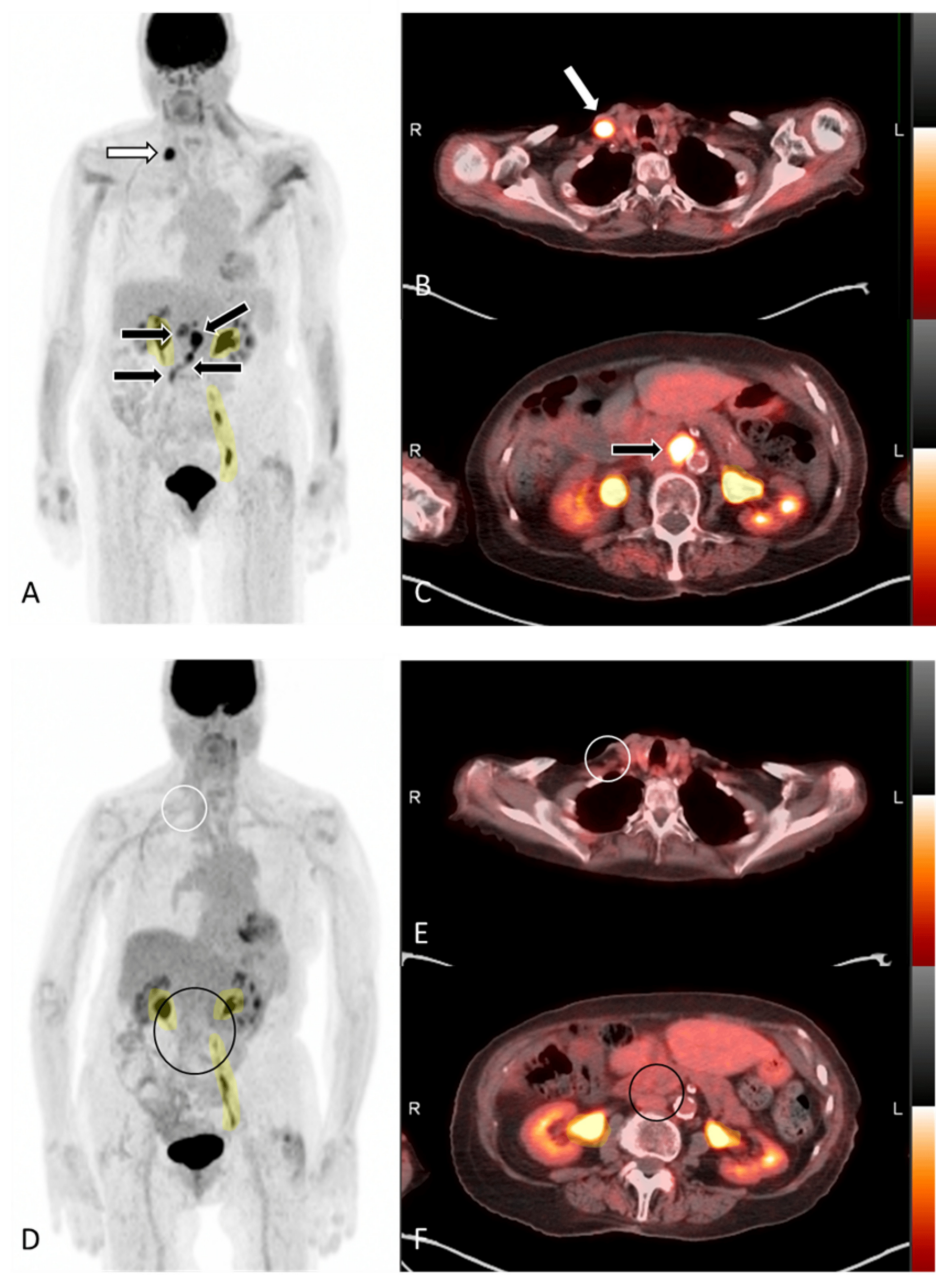
FDG-PET/CT scan FDG-PET/CT demonstrating abscopal effect of distant local radiation on follicular lymphoma. Maximum intensity projection (MIP) (A) and axial fusion (B and C) images from FDG-PET/CT demonstrate FDG-avid lymphadenopathy in the right medial supraclavicular region (white arrows), gastrohepatic region, and retroperitoneum (black arrows). The right supraclavicular lymph node was subsequently biopsied and found to represent grade 3A follicular lymphoma. MIP (D) and axial fusion (E and F) images from six-month follow up FDG PET/CT after distant radiation to the right lower leg for recurrent Merkel cell carcinoma, and without any lymphoma directed or systemic therapy, shows resolution of previously seen FDG-avid lymphadenopathy (white and black circles), consistent with a complete metabolic and anatomic response. Note the shaded yellow areas represent physiologic tracer clearance in the renal collecting system. FDG-PET/CT: fluorodeoxyglucose positron emission tomography/computed tomography

A Caucasian woman in her 90s with a past medical history notable for several non-melanoma skin cancers previously treated with Mohs micrographic surgery, WLE, and intralesional 5-fluorouracil injections presented to the dermatology clinic with a concerning lesion on the dorsum of right foot, which was found to be a primary MCC on shave biopsy (positive IHC for CK20 and negative for TTF-1). Given the high-risk nature of the tumor, patient was urgently referred to oncology and a staging fluorodeoxyglucose positron emission tomography/computed tomography (FDG-PET/CT) scan was performed, which did not show the primary lesion of the right dorsal foot or any regional lymphadenopathy but revealed intense FDG uptake in conglomerate lymph nodes in the gastrohepatic and retroperitoneal stations, which were suspicious for malignancy (Figure 1A-C). 

The patient underwent endoscopic ultrasonography and fine needle aspiration of a few enlarged lymph nodes in the porta hepatis region, which were negative for metastatic MCC or T- or B-cell lymphoproliferative disorders, and so the lymphadenopathy was concluded to be reactive in nature. After discussing the treatment options for her MCC, including surgery, chemotherapy, immunotherapy, and RT, given the patient’s goals of care and overall frailty, a shared decision was made to proceed with WLE alone with no SNL biopsy or adjuvant treatments. Surgical pathology confirmed negative deep and peripheral margins.

**Figure 2:**
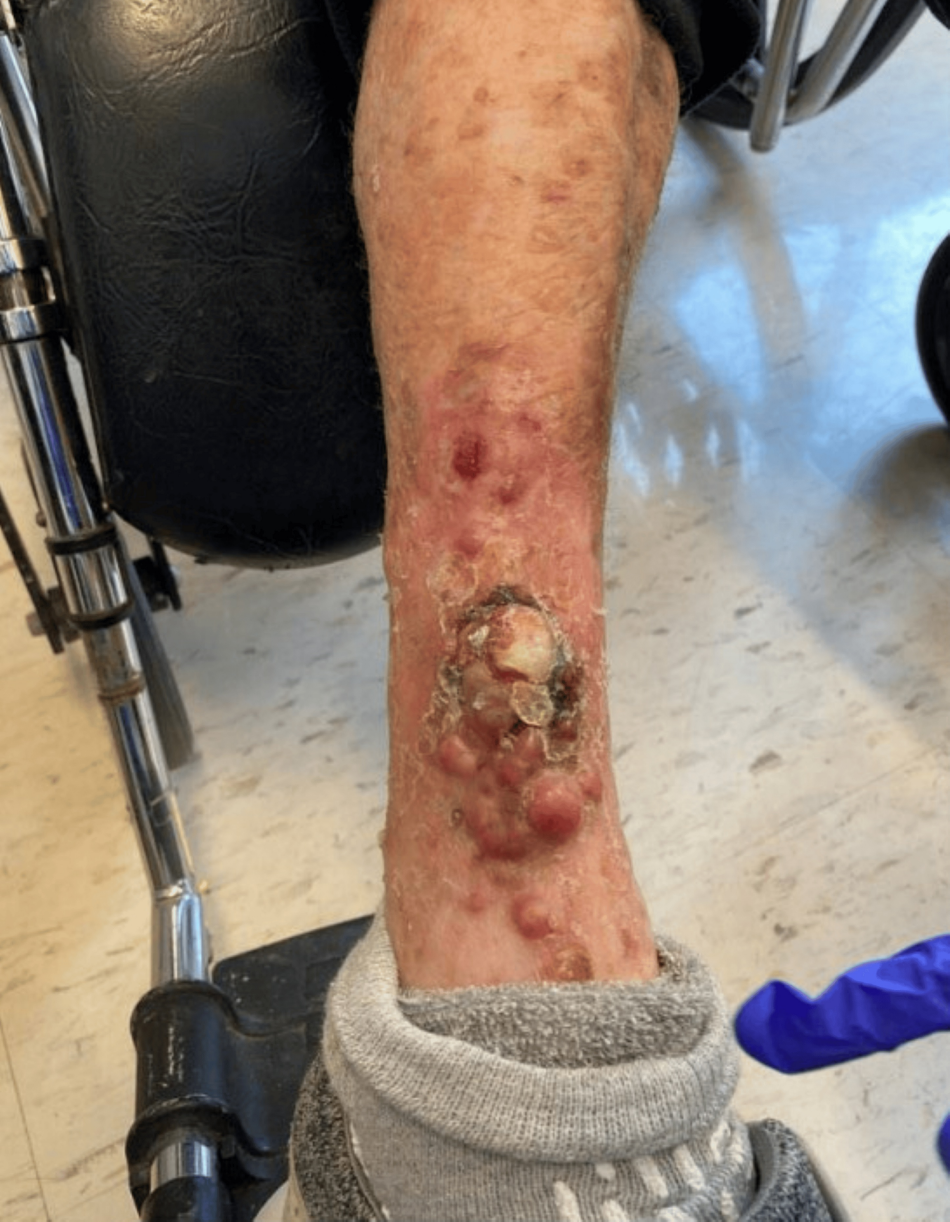
Numerous firm pink exophytic occasionally ulcerated nodules on right shin confirmed to be recurrent or metastatic Merkel cell carcinoma

Three months post-WLE, the patient presented with a new onset of several rapidly growing pink nodules on her right lower shin (Figure 2). Shave biopsy suggested recurrent or metastatic MCC. An interval FDG-PET/CT was performed, which showed multiple FDG-avid subcutaneous nodules along the lateral aspect of the right distal shin, suspicious for in-transit metastasis from the patient's prior MCC on the right dorsal foot. There was also increased size and uptake in multiple retroperitoneal lymph nodes and a new intensely FDG-avid lymph node near the right supraclavicular region was found. Supraclavicular lymph node biopsy was performed and IHC showed CD3- and CD20-positive B-cells that co-expressed CD10, BCL6, BCL2, and CD21, with a variable Ki67 proliferative index of approximately 30%, negative CD30, CK20, and Cyclin D1, with no necrosis or karyorrhectic debris. Flow cytometry revealed a CD10+ kappa restricted monoclonal B cell population, and a diagnosis of grade 3A follicular lymphoma with Follicular Lymphoma International Prognostic Index (FLIPI) score of greater than 3 based on her age, stage III disease, anemia, and multiple areas of lymph node involvement was made.

**Figure 3:**
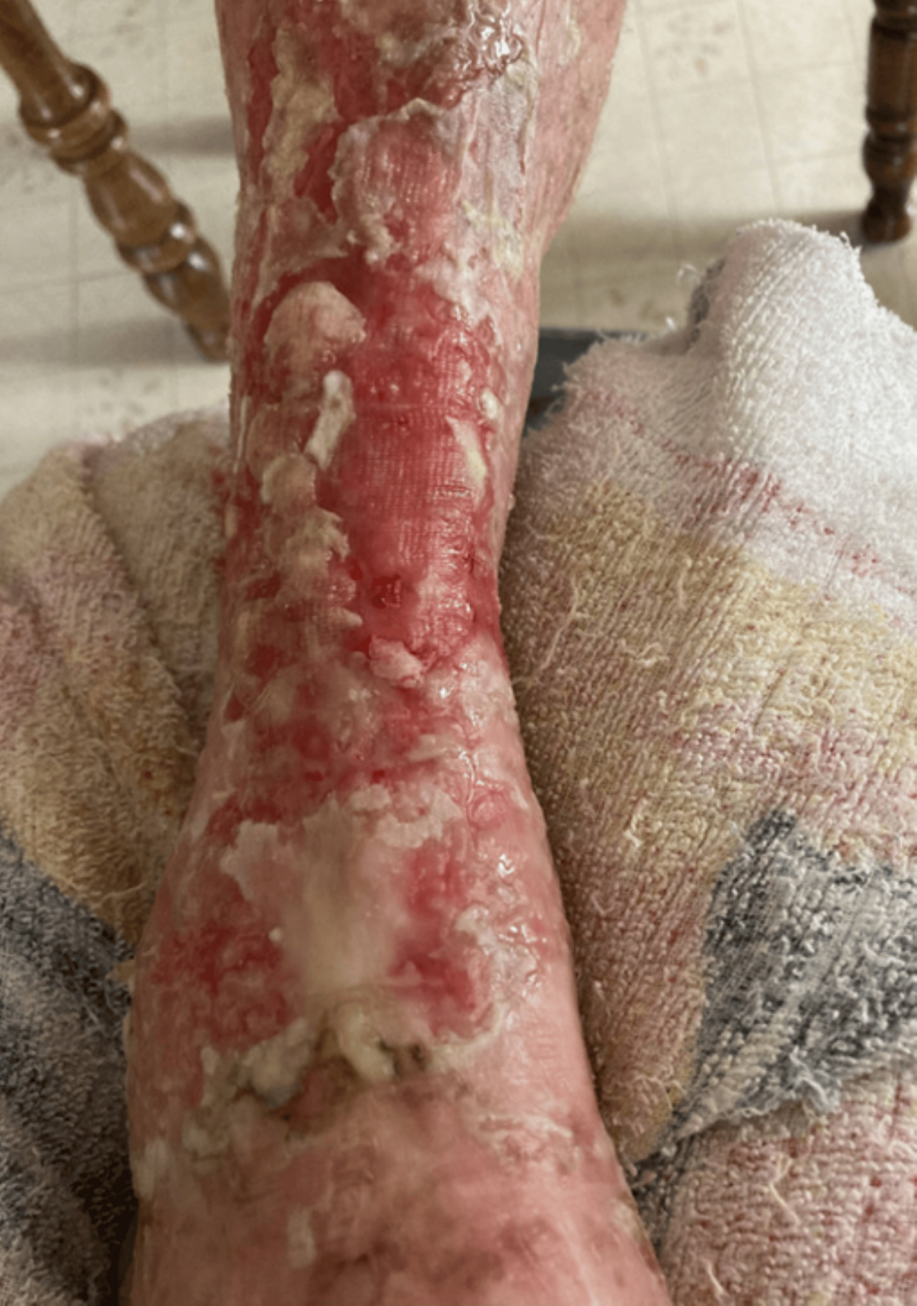
Shallow erosion and diffuse maceration of the right distal shin and anterior ankle suggestive of gradual resolution of the Merkel cell carcinoma after a two-month course of radiation therapy

**Figure 4:**
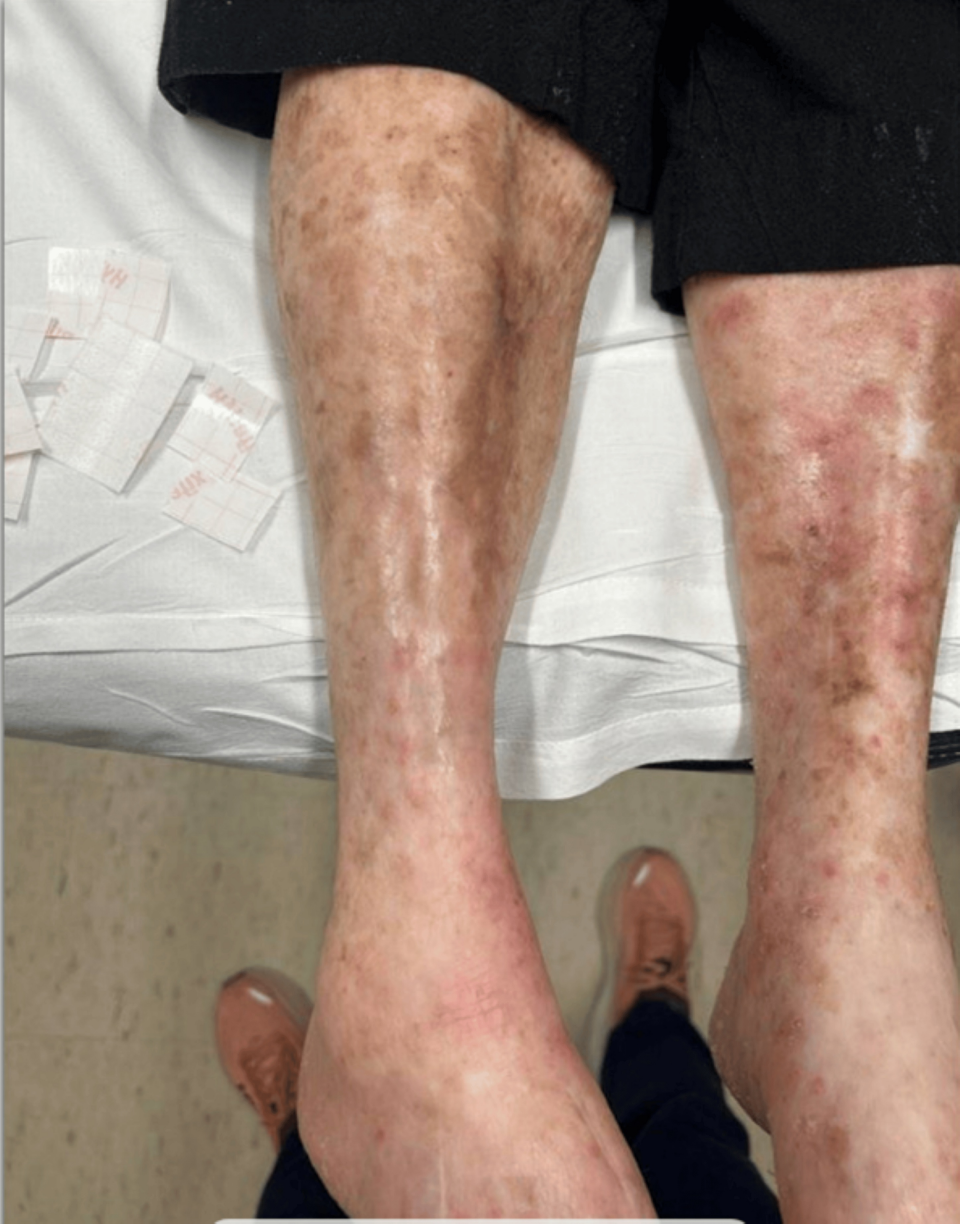
Significant reduction and focal resolution of the biopsy proven recurrent Merkel cell carcinoma on the right shin three months after finishing radiation therapy

Given the relatively low-grade nature of her lymphoma (two-year overall survival of 87% per her FLIPI score), treatment of her recurrent MCC was prioritized and so she underwent RT of the right shin, which led to the gradual improvement (Figure 3) and almost complete resolution (Figure 4) of her MCC after receiving a total of 6700 cGy for five days a week over a period of two months. A repeat FDG-PET/CT at three months post-RT (six months post-WLE) was notable for complete metabolic and anatomic response of the MCC on the right shin with a score of 1 based on Deauville five-point scoring system, showing no uptake above background. Perhaps more notably, however, complete resolution of the previously seen FDG-avid lymph nodes in the abdomen and supraclavicular regions (Figure 1D-F) were also noted. The MCC on the right shin unfortunately recurred and the patient underwent repeat WLE, but her follicular lymphoma has since remained in remission.

## Discussion

MCC is a rare and aggressive form of neuroendocrine cancer. Due to its high metastatic potential, MCC is usually treated with a combination of surgery, immunotherapy, and RT. Despite aggressive treatment of the primary malignancy, however, MCC may recur or metastasize. Follicular lymphoma, on the other hand, is an indolent hematogenous malignancy and, depending on its stage and tumor burden, can be treated with a combination of RT, chemotherapy, or immunotherapy. While previous reports of spontaneous resolution of follicular lymphoma in response to uninvolved-site RT targeting FL are available, the order of events in our patient favor an abscopal effect, which to our knowledge has not been previously reported in the literature [16]. An alternative hypothesis would be the priming of the immune system following the supraclavicular lymph node biopsy, which could have exposed follicular lymphoma-specific neoantigens to the circulating B- or T-cells, leading to the follicular lymphoma's systemic destruction. Another hypothesis, although less favored, is that her follicular lymphoma underwent spontaneous regression independent of her concurrent MCC treatments - a phenomenon that has been rarely reported in the literature [12]. The priming of the immune system in the setting of recurrent MCC may have also played a role in independently clearing the follicular lymphoma via promoting a non-specific, pro-inflammatory state.

Although the exact mechanism of the abscopal effect is not well understood, it is thought to be mediated by a complex process involving the activation of the adaptive and the innate immune systems [16,17]. Irradiation of a malignant cell results in the release of damage-associated molecular patterns (DAMPs) such as radiation-induced damaged DNA, mitochondrial peptides, or calreticulin [18]. Cytotoxic CD8+ T cells, which are the major players in the adaptive immune system and can recognize DAMPs, have been shown to be strongly associated with the abscopal effect [17,19]. Furthermore, the innate immunity, particularly the dendritic cells and M1 macrophages in the tumor microenvironment, have also been shown to contribute to the abscopal effect in breast cancer and in situ studies [16,17]. Conversion of anti-inflammatory M2 to pro-inflammatory M1 macrophages upon irradiation leads to upregulation of tumor necrosis factor (TNF)-α, enhancing the anti-tumor activity of the innate immune system as well. Furthermore, the bone marrow has been shown to produce more pro-inflammatory cytokines such as TNF-α as well as CD95L+ (FasL+) cytotoxic T cells and natural killers upon irradiation [17,20].

If the resolution of this patient's follicular lymphoma following the RT of her MCC was due to an abscopal effect, several hypotheses may be considered as to why follicular lymphoma, which is a seemingly unrelated malignancy, could have responded to uninvolved site RT in this patient. One hypothesis is the formation of DAMPs in response to RT of the MCC, which may have been similar enough to the tumor-associated antigens in her follicular lymphoma that could be recognized by the activated T-cells, leading to their destruction. Another, although less favored, hypothesis is presence of follicular lymphoma in the irradiated field on the patient's shin, leading to an abscopal effect irrespective of her MCC, which is less likely given the findings of the PET scans.

Further in vivo and in vitro studies are needed to better understand the underlying molecular mechanisms in the abscopal effect seen in this patient. One approach may be to perform a comparative proteomic analysis of irradiated and non-irradiated MCC and follicular lymphoma to identify molecular mimickers shared between the different entities. Additionally, T-cell affinity studies may be performed to investigate any overlap between the T-cell activation patterns in MCC and follicular lymphoma. Lastly, retrospective or prospective cohort studies using the Merkel Cell Carcinoma Patient Registry may be performed to further study the abscopal effect in this patient population.

Our patient was delighted about the resolution of her follicular lymphoma after RT and continues to work with her multidisciplinary team to manage her recurrent MCC.

## Conclusions

The abscopal effect is an immunological phenomenon that has been previously reported in the context of uninvolved site radiation and resolution of malignancies such as follicular lymphoma. To our knowledge, however, this phenomenon has not been previously reported in the context of uninvolved site radiation of a cancerous growth (i.e. MCC) and complete metabolic and anatomic resolution of another malignancy (i.e. follicular lymphoma), making this a novel case report, although alternative hypotheses exist that may be able to explain this observation. Additional studies, such as proteomic analysis and T-cell affinity studies, are needed to better understand the underlying molecular mechanism in the abscopal effect and to investigate the plausibility of this phenomenon in concurrently occurring malignancies.
